# Multi-View Gait Analysis by Temporal Geometric Features of Human Body Parts

**DOI:** 10.3390/jimaging10040088

**Published:** 2024-04-09

**Authors:** Thanyamon Pattanapisont, Kazunori Kotani, Prarinya Siritanawan, Toshiaki Kondo, Jessada Karnjana

**Affiliations:** 1 School of Information Science, Japan Advanced Institute of Science and Technology, Nomi 923-1211, Japan; s2120416@jaist.ac.jp (T.P.); ikko@jaist.ac.jp (K.K.); 2 School of Information, Computer, and Communication Technology, Sirindhorn International Institute of Technology, Pathum Thani 12120, Thailand; tkondo@siit.tu.ac.th; 3 National Electronics and Computer Technology Center, National Science and Technology Development Agency, Pathum Thani 12120, Thailand; jessada.karnjana@nectec.or.th

**Keywords:** multi-view gait analysis, correlation feature, dynamic time warping, voting algorithm

## Abstract

A gait is a walking pattern that can help identify a person. Recently, gait analysis employed a vision-based pose estimation for further feature extraction. This research aims to identify a person by analyzing their walking pattern. Moreover, the authors intend to expand gait analysis for other tasks, e.g., the analysis of clinical, psychological, and emotional tasks. The vision-based human pose estimation method is used in this study to extract the joint angles and rank correlation between them. We deploy the multi-view gait databases for the experiment, i.e., CASIA-B and OUMVLP-Pose. The features are separated into three parts, i.e., whole, upper, and lower body features, to study the effect of the human body part features on an analysis of the gait. For person identity matching, a minimum Dynamic Time Warping (DTW) distance is determined. Additionally, we apply a majority voting algorithm to integrate the separated matching results from multiple cameras to enhance accuracy, and it improved up to approximately 30% compared to matching without majority voting.

## 1. Introduction

**Figure 1:**
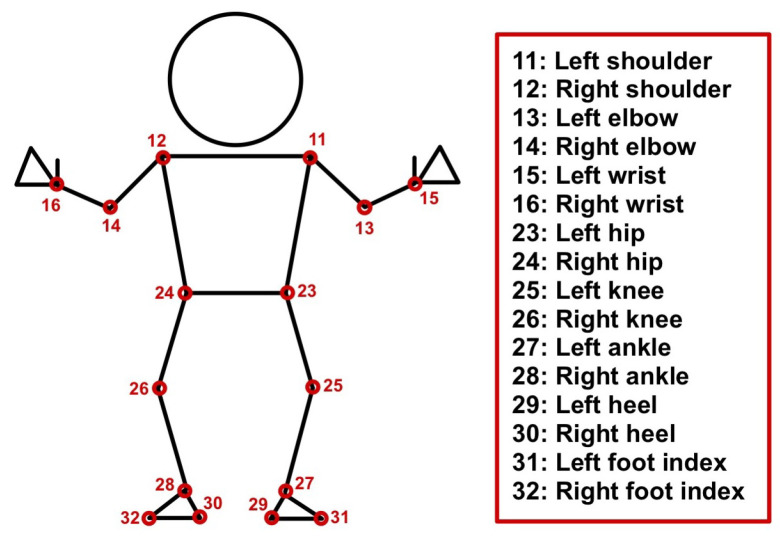
Sixteen keypoints from MediaPipe pose estimation used in this study.

Gait is an individual’s walking pattern that involves position changes in the upper and lower body. In other words, it refers to the movement of a joint as it changes position over time. In recent years, vision-based joint estimation has been widely deployed. We let $p_{i}^{j}\left(x,y,z,t\right)$ represent a joint number *j* in Figure 1, which presents the human body joints from MediaPipe [1] that was used in this study. *i* represents a person index, *x*, *y*, and *z* represent the *x*, *y*, and *z* axes of the joint coordinates, and *t* represents time. The changing of $p_{i}^{j}\left(x,y,z,t\right)$ can present a walking pattern. It can represent the personality of a person, e.g., identity, emotions, health, and more [2].

### 1.1. Gait Analysis

Human gait can be applied to analyze neurological disorders, such as Parkinson’s disease. It is a progressive disorder that affects the nervous system, and some symptoms are reflected in the walking pattern. The research by S. R. Hundza et al. [3] presented the Inertia Measurement Unit (IMU)-based gait cycle detection in Parkinson’s disease (PD) using gyroscope angular rate reversal to address the initial gait cycle of PD test subjects. A. P. Rocha et al. [4] employed the Kinect RGB-D camera system as a tool to assess PD by extracting the skeleton of PD patients. Their target was to distinguish between PD and non-PD subjects and between two PD states. G. Sun and Z. Wang [5] employed vision-based fall detection by OpenPose to figure out the human pose and conducted SSD mobilenet object detection to get rid of OpenPose’s mistakes and applied SVDD classification for fall detection. D. Slijepcevic et al. [6] used different machine learning (ML) and deep neural network (DNN) techniques to classified the walking patterns of children who have Celebral Palsy (CP). They aimed for explainable ML to gain trust for using it to analyze human gait. They found that the classification from ML approaches is better than DNNs. However, DNNs employed additional features to predict the results. Some previous works show that emotion detection by using human gait was possible, as per the survey by S. Xu et al. [7]. In their study, G. E. Kang et al. [8] examined how bipolar disorder patients control their balance while walking and sitting to walk. They used motion data from 16 cameras to achieve this. Moreover, N. Jianwattanapaisarn [9] proposed the analysis of an emotion characteristic by prompting 49 subjects to walk in a setting region while watching the emotion-inducing videos on Microsoft Hololens 2 smart glasses. They used OptiTrack motion capture to obtained human gaits and postures and extracted features such as the angle between body parts and walking straightness for analysis.

Different types of methods were used to study gait, including non-training-based methods like Dynamic Time Warping (DTW), Decision Tree, K-means clustering, and Support Vector Machine (SVM), as well as training-based methods like CNN, Grad-CAM, and Long Short-Term Memory (LSTM). In fact, all of the algorithms are reliable, but the non-training-based DTW is selected for this study because it is an uncomplicated method for matching the patterns. Since it is a classification technique, it does not require data training or a large number of datasets. R. Hughes et al. [10] improved the floor-based monitoring system and implemented DTW with KNN to enhance walking identification. M. Błażkiewicz et al. [11] applied DTW to assess the gait asymmetry of barefoot walking to evaluate the gait symmetry. The work by Y. Ge et al. [12] employed DTW to match the signals from LoRa sensors with a database to recognize the gait. D. Avola et al. proposed wearable sensor-based gait recognition using a smartphone accelerometer, based on a modified DTW, and applied modified majority voting to return the matched identity of the best comparison score to improve the recognition’s accuracy [13]. Previous works show that non-training-based methods are effective in recognizing gait. However, training-based methods are essential for tasks beyond recognition. Hence, we intend to implement the training-based method for extending the gait analysis tasks.

### 1.2. Gait Recognition

There are two main approaches for gait recognition, i.e., appearance-based and model-based approaches [2]. The appearance-based approach is model-free; it bases analysis directly on silhouette sequences to deploy the shape and textural information as features for gait analysis. The following studies present gait recognition based on a person’s appearance. The work by M. Alotaibi and A. Mahmood [14] intended to increase gait recognition accuracy by developing eight layers of deep CNN that are less sensitive to variations and occlusions. They employed CASIA-B, a multi-view gait database with various walking conditions, for the experiment. Their proposed method can overcome several issues, but performance decreases if the gallery set does not cover a variety of walking conditions. They achieved an average correct classification, rank-1, and rank-5 accuracy of 86.70%, 85.51%, and 96.21% on the CASIA-B dataset, respectively. M. Deng and C. Wang focused on proposed gait recognition in different clothing conditions [15]. They employed silhouette gait images to extract the shape of a human and divide it into four sub-regions. Then, they selected the gait features based on the width of each sub-region and input the gait feature vector into Radial Basis Function (RBF) neural networks. Their proposed method gave the correct classification rate on NM (normal walking) and CL (walking while wearing a down coat) conditions of the CASIA-B dataset at 90% when using NM as a probe set and at 93.5% when using the CL condition as a probe set. S. Hou et al. developed the Gait Lateral Network (GLN) to recognize the human gait [16]. It is a deep CNN that can learn discriminative and compact representations from silhouette images. GLN achieved an average rank-1 accuracy of 96.88% on NM and 94.04% on a BG (walking while carrying a bag) condition of the CASIA-B dataset, respectively. However, the clothing condition affects the slight decrease in rank-1 accuracy to 77.50%. C. Fan et al. [17] claimed that different parts of the human body consist of diverse visual appearances and movement patterns during walking. GaitPart was proposed as a way to extract gait features. The goal was to improve the learning of part-level features using a frame-level part feature extractor made up of FConv and obtain the short-range spatiotemporal expression using a Temporal Feature Aggregator with a Micromotion Capture Module (MCM). The results from GaitPart achieved average rank-1 accuracy on the CASIA-B dataset of 96.2% on NM, 91.5% on BG, 78.7% on CL conditions, and 88.7% on the OU-MVLP dataset. GaitEdge was a framework described by J. Liang et al. [18] for recognizing human gait. It made this framework more practical and kept performance from dropping in cross-domain situations by blocking irrelevant gait information. They designed the module to integrate the trainable edges of the segmented person’s shape with the fixed internals of silhouette images based on the mask operation, named Gait Synthesis. GaitEdge achieved an average rank-1 accuracy on the CASIA-B* dataset (across different views) of 97.9% on NM, 96.1% on BG, and 86.4% on CL conditions.

Our research interest is the model-based approach. It requires a mathematical model to distinguish the gait characteristics. The earlier works from R. Liao et al. [19] proposed a model-based gait recognition by extracting 14 body joints of 2D human pose estimation from images and transforming them into 3D poses, called PoseGait. The CNN is implemented to extract the gait features. Moreover, they combined three spatio-temporal features with the body pose to enhance the features and recognition rate. Their proposed method achieved recognition rates on the CASIA-B dataset of 63.78% on NM, 42.52% on BG, and 31.98% on CL conditions. Additionally, they proposed another model-based method for gait recognition with pose estimation and graph convolutional networks, named PoseMapGait [20]. They aimed to preserve the robustness against human shape and the human body cues of the gait feature by using a pose estimation map, which claims to enrich the recognition rate. PoseMapGait achieves the average recognition rate on the CASIA-B dataset of 75.7% on NM, 58.1% on BG, and 41.2% on CL conditions. X. Li et al. [21] mentioned the information loss suffering of 2D poses, unlike 3D poses. They presented a 3D human mesh model with parametric pose and shape features. In addition, they trained a multi-view to overcome the poor pose estimation in a 3D space. They achieved rank-1 accuracy on the CASIA-B dataset of 60.92% on NM, 42.01% on BG, and 32.81% on CL conditions. This study was not trained for gait recognition directly, but the authors aimed to create the database for multiple related purposes. The research from C. Xu et al. [22] considered the occlusion-aware human mesh model for gait recognition. They mentioned that partial occlusion of the human body mostly occurs in surveillance scenes. So, they created model-based gait recognition for handling the occluded gait sequences without any prerequisite. They set the SMPL-based human mesh model to an input image directly, and extracted the pose and shape features for the recognition task. The most challenging part was when the occluded ratio was huge (around 60%). Their proposed method outperformed the other state-of-the-art methods by 15% of rank-1 accuracy. K. Han et al. proposed a discontinuous gait image recognition based on the extracted keypoints of the human skeleton [23]. They aimed to overcome the situation of discontinuity in the gait images. This study achieved a high recognition rate and is robust to common variations. Mostly, model-based gait analysis aimed to increase the recognition rate by implementing machine learning. They achieve the average rank-1 accuracy on the three conditions of the CASIA-B dataset of 79.5%.

Previous studies have addressed variations such as camera perspective, clothing, illumination, occlusion, and carrying that make gait analysis unreliable. These variations are a significant challenge for analyzing the gait. Additionally, it is essential to apply gait analysis in practical settings where equipped stationary cameras whose perspectives are limited, such as surveillance cameras, unlike in laboratory settings.

### 1.3. Vision-Based Human Pose Estimation

The authors extract features as joint angles based on human joint estimation by Google MediaPipe [1]. It uses the BlazePose model, a lightweight model that produces thirty-three keypoints of pose estimation, but we employ only sixteen keypoints for the experiment, as shown in Figure 1. Each keypoint contains the coordinates in the *x*, *y*, and *z* axes, unlike the OpenPose [24] and AlphaPose [25] that produce 18 keypoints of the estimated joints in the *x* and *y* axes. However, MediaPipe is only a single-person pose estimation, while both OpenPose and AlphaPose are multi-person pose estimations. Since most datasets provide sequences with a single walker, a single-person pose estimation is sufficient. A previous study from X. Li et al. [26] proposed fitness action counting and classification based on MediaPipe. They presented the comparative results between MediaPipe, OpenPose, and Alphapose, which claimed that MediaPipe is faster to recognize and achieved high accuracy. K. Y. Chen et al. used MediaPipe to obtain the features, and employed transfer learning deep neural networks to determine the type of fitness movement and its completeness [27]. The authors also suggested that MediaPipe has an uncomplicated implementation, fast computational speed, and high accuracy.

This study proposes person identification by majority voting based on DTW matching. It is walking pattern matching, which is a non-training-based approach. Once we extract the human pose landmarks, we use the Euclidean distance to create a triangle. Then, we apply the cosine law to extract the desired joint angles based on the performed triangle, i.e., elbow, hip, knee, and ankle angles, and calculate rank correlation between the extracted joint angles. We deploy two features, i.e., joint angles and correlation, for measuring the DTW distance. Finally, we match people in each camera perspective of the target sequences and apply a majority vote to increase the matching results. We divide features into three parts corresponding to the upper, lower, and whole body to study the effect of walking patterns. Additionally, we divide the number of subjects into three cases to observe the results of the proposed method.

Our research goal is to analyze human motion from a multi-view gait image for human behavior analysis based on their walking pattern and to expand the range of our research to include clinical, psychological, and effective analysis tasks in addition to recognition tasks.

Let us address our highlights and the advantages of the research as follows:

- We implement Dynamic Time Warping (DTW) to match the walking patterns.
- We deploy the joint angles and the rank correlation between each angle in parallel as features for measuring the DTW distance.
- A majority vote is applied to increase the matching performance over multiple cameras.
- Small datasets can be employed by the proposed method, which is a non-training-based approach.
- Detailed analyses are possible due to the availability of data visualization.
- The proposed method is implemented on the CPU, which has advantages in terms of time and cost savings.

## 2. Materials and Methods

This section explains our proposed method for multi-view person’s identity matching based on DTW and the majority voting algorithm. The joint angles and their correlation are determined to be employed as features for a matching purpose.

### 2.1. Methodology

**Figure 2:**
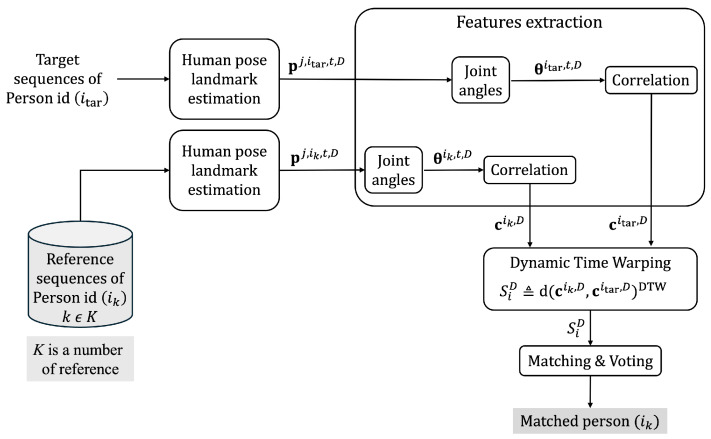
Overall methodology of this study.

Figure 2 shows the overall methodology of this study. The authors extract the joint coordinates of the reference and target sequences as $p^{j,i_{k},t,D}$ and $p^{j,i_{tar},t,D}$. Variable *k* represents the number of reference sequences, which is kϵK. We let *j* be a joint number; *D* is a camera perspective, and *i* is a person identity label. Then, we extract the features from reference and target sequences as the joint angles ($\theta^{i_{ref},t,D}$ and $\theta^{i_{tar},t,D}$), which consist of 10 angles, including the elbow, hip, knee, and ankle (front and back), on both the left and right sides of the body. Next, we calculate the rank correlation between each feature vector of reference and target sequences as $c^{i_{ref},t,D}$ and $c^{i_{tar},t,D}$. Then, we apply Dynamic Time Warping (DTW) to measure the distance between them as $S_{i}^{D}$. After that, we find a minimum DTW distance to match a person’s identity in the target sequence with the reference sequence. The matched person identity is returned as $i_{k}^{D}$, which refers to the person identity in each camera perspective (*D*). Finally, we aggregate the separated person identity ($i_{k}^{D}$) from each *D* by applying majority voting to increase the reliability of a person identity matching.

### 2.2. Joint Angles Calculation

**Figure 3:**
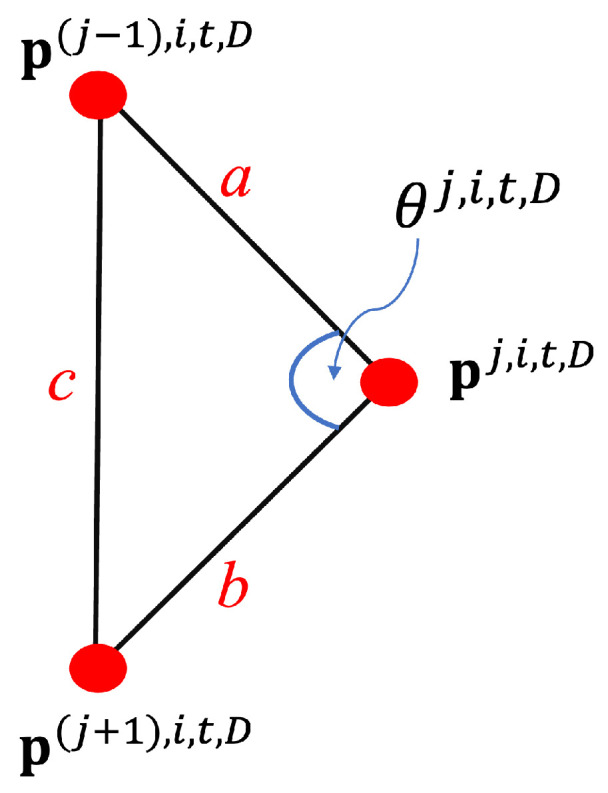
Joint angle calculation using cosine law to calculate a middle angle ($\theta^{j,i,t,d}$) between 3 joints.

To extract the joint angles, we initialize the process by connecting 3 joints, i.e., $p^{(j-1),i,t,D}$, $p^{j,i,t,D}$, and $p^{(j+1),i,t,D}$, from Google MediaPipe in Figure 1 as a triangle. Figure 3 represents the mentioned triangle connection. We apply the Euclidean distance between each joint, which is determined to connect them for generating a triangle. We let it be Legs *a*, *b*, and *c* using Equations (1)–(3). Leg *a* represents a connection line between joints $p^{(j-1),i,t,D}$ and $p^{(j),i,t,D}$, Leg b is a connection line between joints $p^{j,i,t,D}$ and $p^{(j+1),i,t,D}$, and Leg *c* is a connection line between joints $p^{(j-1),i,t,D}$ and $p^{(j+1),i,t,D}$. Finally, we apply the cosine law in Equation (4) to extract the middle angle ($\theta^{j,i,t,D}$), which is a preferred joint angle to use as a feature.

(1) $$a=textbfp^{j,i,t,D}-p^{(j-1),i,t,D}\;$$

(2) $$b=textbfp^{j,i,t,D}-p^{(j+1),i,t,D}\;$$

(3) $$c=textbfp^{(j-1),i,t,D}-p^{(j+1),i,t,D}\;$$

(4) $$\theta^{j,i,t,D}=\cos^{-1}\left(\frac{\left(a^{2}+b^{2}\right)-c^{2}}{2\times (\sqrt{a^{2}}\times \sqrt{b^{2}})}\right)$$

This study separates feature vectors into three parts, i.e., whole, upper, and lower body, to determine DTW distance for a matching purpose. Typically, human gait refers to the motion of lower body parts, i.e., hip, knee, and ankle. However, we notice that the whole body has motion while humans walk, not just the lower parts. Thus, we decide to employ the upper body feature to study the effect of the body parts on an analysis of the gait in various walking conditions.

The $\theta^{i,t,D}$ value in Equation (5) represents a feature vector of the whole body consisting of 10 angles. Upper body ($\theta_{u}^{i,t,D}$) consists of 2 angles, left and right elbow, as in Equation (6). The lower body ($\theta_{l}^{i,t,D}$) consists of 8 angles, left and right hip, knee, and ankle (front and back), as in Equation (7).

(5) $$\theta^{i,t,D}={\left[\theta^{1,i,t,D}\theta^{2,i,t,D}\ldots \theta^{j,i,t,D}\ldots \theta^{10,i,t,D}\right]}^{T}$$

(6) $$\theta_{u}^{i,t,D}={\left[\theta^{1,i,t,D}\theta^{2,i,t,D}\right]}^{T}$$

(7) $$\theta_{l}^{i,t,D}={\left[\theta^{3,i,t,D}\theta^{4,i,t,D}\ldots \theta^{j,i,t,D}\ldots \theta^{10,i,t,D}\right]}^{T}$$

### 2.3. Correlation Calculation

In this study, we calculate the correlation between joint angles, treating them as individual patterns that serve as features for matching. Based on frame-by-frame human pose extraction, we extract individual joint angles with respect to the frame, resulting in a pattern that is frame-dependent. The rank correlation aims to be a frame-independent feature and it can provide more stability and enhance the reliability of the matching. Moreover, when a person is walking, their whole body is moving. Thus, all joints are correlated.

Spearman correlation is a method to measure dependence between two ranking variables [28]. It is a non-parametric rank measurement that employs a monotonic function to define a relationship between them. The calculated correlation is [−1, 1], which implies that two variables are similar and have a positive monotonic relationship when it is closer to 1. However, if it is closer to −1, the two variables are perfectly opposite and have a negative monotonic relationship. There is no correlation if a calculated value is around 0.

**Figure 4:**
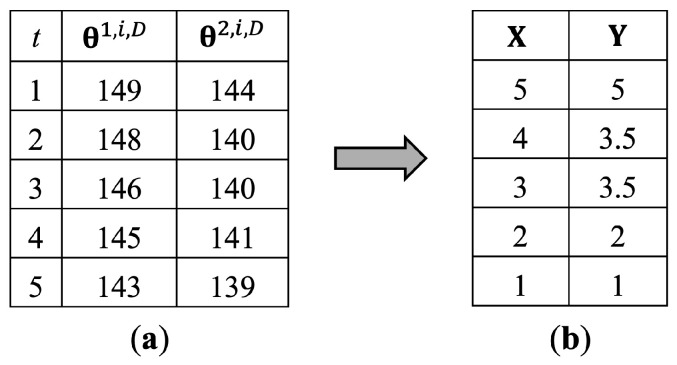
Sample ranking of $\theta^{1,i,D}$ and $\theta^{2,i,D}$ for calculating the correlation between them. (**a**) is the values before ranking of $\theta^{1,i,D}$ and $\theta^{2,i,D}$. (**b**) is the values after ranking of $\theta^{1,i,D}$ (**X**) and $\theta^{2,i,D}$ (**Y**).

Figure 4 shows the way to assign the ranks to $\theta^{1,i,D}$ and $\theta^{2,i,D}$ values. The $\theta^{j,i,D}$ value represents values of joint angle *j* in every *t*, as shown in Equation (8). Figure 4a presents the values of $\theta^{1,i,D}$ and $\theta^{2,i,D}$ before assigning the ranks, and Figure 4b presents the rankings of $\theta^{1,i,D}$ and $\theta^{2,i,D}$ as **X** and **Y**, respectively. The values are arranged from minimum to maximum, assigning a minimum value to the first order. For the tied ranks, the average number between them will be assigned to all tied ranks. Figure 4a shows that there are two identical values of $\theta^{2,i,D}$, which actually are orders of 3 and 4, but we assign an order of 3.5 as they are tied ranks.

This study calculates an individual correlation between each joint angle as a sample in Table 1 and applies it as a feature for pattern matching. Each correlation feature is a 2D array that consists of ten 1D arrays inside, as each row of the table in Table 1. It is an individual walking pattern in terms of the correlation between joint angles.

(8) $$\theta^{j,i,D}={\left[\theta^{j,i,1,D}\theta^{j,i,2,D}\ldots \theta^{j,i,t,D}\right]}^{T}$$

Equation (9) presents the correlation calculation in this study. The E[•] value represents an expected value of vectors **X** and **Y**. The correlation between angles of reference and target sequences is calculated separately as $c^{i_{ref},D}$ and $c^{i_{tar},D}$.

(9) $$c^{i,D}=\frac{E[XY]-E[X]E[Y]}{\sqrt{E\left[X^{2}\right]-E{\left[X\right]}^{2}}\sqrt{E\left[Y^{2}\right]-E{\left[Y\right]}^{2}}}$$

### 2.4. Distance Measurement

Dynamic Time Warping (DTW) is an algorithm to measure the distance between time series which can be used to find similarities. This algorithm can handle varying walking speeds and endure time shifts between two sequences. This algorithm is versatile and can be used for different recognition tasks, such as speech and signature recognition, as in the work of C. S. Myers and L. R. Rabiner [29].

**Figure 5:**
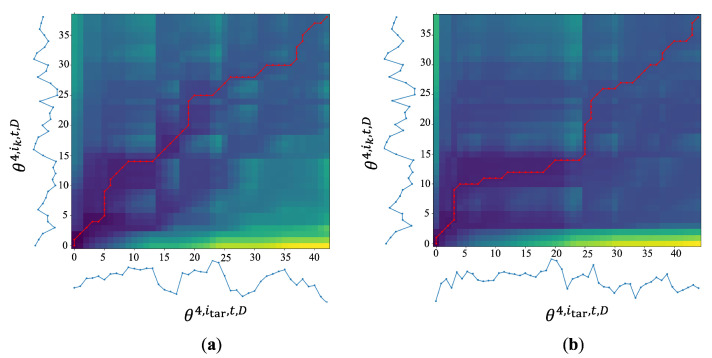
Sample of the DTW warping path (red line) on the cost matrix shown as a heat map of right hip angle at *D* = $162^{\circ }$. (**a**) DTW warping path with the same person. (**b**) DTW warping path with a different person.

DTW offers the most affordable and optimum option for two sequences to be aligned, known as the DTW distance ($S_{i}^{D}$). Figure 5 shows an example of the DTW warping path on the cost matrix of the right hip angle of reference (*y*-axis) and target sequences (*x*-axis) between the same person (Figure 5a) and a different person (Figure 5b). It indicates that the warping path of the same person is diagonal from the starting point to the endpoint, unlike the warping path of the different person. The more straight warping path refers to an optimal path, which implies both patterns require a lower cost to be aligned. Since DTW can be one-to-multiple alignment, the more diagonal paths, the greater the similarity between the two patterns.

We employ features from Equations (5)–(9) as the features for determining DTW distance as $S_{i}^{D}$, and then multi-dimensional DTW is implemented. The dependent DTW is applied to calculate the distance between two multi-dimensional models. In the research of M. Shokoohi-Yekta et al. [30], it is called ${\mathrm{DTW}}_{D}$. It warps all dimensions into a single matrix as a single-dimensional DTW calculation and calculates the distance between two matrices.

### 2.5. Matching Algorithm and Voting

After determining DTW distance, we match the person identity in a target with reference sequences by finding a minimum DTW distance as in Equation (10). Since it has multiple cameras for multi-view gait analysis, we let $i_{k}^{D}$ represent the matched person identity from each camera perspective (*D*).

(10) $$i_{k}^{D}=\underset{i_{k}}{\mathrm{argmin}}\left(S_{i}^{D}\right)$$

Since the multi-view databases use multiple cameras, we obtain multiple matched identities. This implies that the accuracy of the matching depends on the camera perspective. We then apply majority voting to aggregate the identity from each *D* by selecting the most frequently appearing identity in every view. The ’vote’ function in Equation (11) refers to the mentioned voting algorithm. In fact, it is simply a mode in statistics [31].

(11) $$i_{k}=\mathrm{vote}\left\{i_{k}^{0^{\circ }},\ldots ,i_{k}^{D},\ldots ,i_{k}^{180^{\circ }}\right\}$$

## 3. Results

We evaluate the proposed method with CASIA-B [32] and OUMVLP-pose [33] datasets by calculating the accuracy in Equations (12) and (13). It is used to measure the correctness of the matched identities.

(12) $$\mathrm{Accuracy}\;(\mathrm{without}\;\mathrm{voting})=\frac{\mathrm{Total}\;\mathrm{number}\;\mathrm{of}\;i_{k}^{D}}{\mathrm{Total}\;\mathrm{number}\;\mathrm{of}\;\mathrm{true}\;\mathrm{label}}$$

(13) $$\mathrm{Accuracy}\;(\mathrm{with}\;\mathrm{voting})=\frac{\mathrm{Total}\;\mathrm{number}\;\mathrm{of}\;i_{k}}{\mathrm{Total}\;\mathrm{number}\;\mathrm{of}\;\mathrm{true}\;\mathrm{label}}$$

We separate the features into three parts, i.e., the whole, upper, and lower body parts. In this study, we choose the identical view case because the proposed method is a pattern-matching method without a learning state, in contrast to a training-based approach. We apply a majority vote to enhance the reliability of the multi-view walking pattern matching instead. In addition, we experiment with various sample sizes to show the performance of the proposed method.

### 3.1. CASIA-B Datset

**Figure 6:**
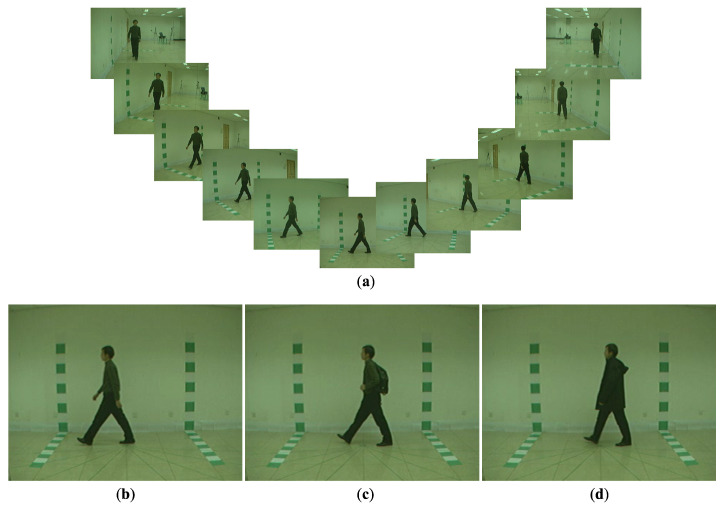
Samples of a multi-view CASIA-B gait database [32]. (**a**) Gait images from the different camera perspectives. (**b**) Normal walking condition (NM dataset). (**c**) Walking with carrying condition (BG dataset). (**d**) Walking with clothing condition (CL).

We apply our method to the CASIA-B dataset [32]. It is a multi-view gait database that captures 124 subjects with 11 cameras, as shown in Figure 6a. The camera perspective spans from $0^{\circ }$ to $180^{\circ }$ with $18^{\circ }$ intervals. All sequences capture the subjects walking from a starting point to the marked endpoint. The $0^{\circ }$ captures a frontal perspective, the $90^{\circ }$ captures a side perspective, and the $180^{\circ }$ captures a rear perspective. There are three walking conditions, i.e., normal walking (NM), walking while carrying a shoulder bag (BG), and walking while wearing a down coat (CL), as shown in Figure 6b–d. The NM condition consists of six sub-datasets. The BG and CL conditions consist of two sub-datasets per each. This study employs 20, 50, and 118 subjects from CASIA-B with two sub-datasets per condition (one as a reference and one as a target for matching) for experimenting. We apply MediaPipe to extract a human skeleton.

**Figure 7:**
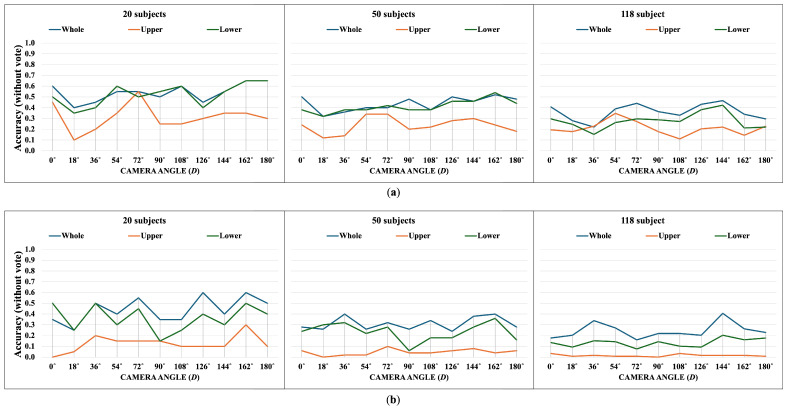
Accuracy of the matching without majority voting on NM condition. (**a**) Accuracy of the joint angles being used as a feature of 20, 50, and 118 subjects. (**b**) Accuracy of the correlation being used as a feature of 20, 50, and 118 subjects.

**Figure 8:**
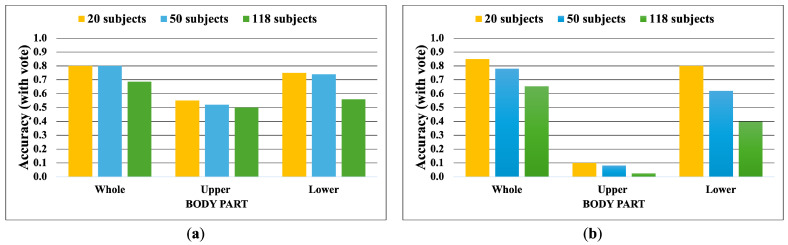
Accuracy of the matching with a majority vote on NM condition. (**a**) Accuracy with majority voting of the joint angles being used as a feature of 20, 50, and 118 subjects. (**b**) Accuracy with majority voting of the correlation being used as a feature of 20, 50, and 118 subjects.

**Figure 9:**
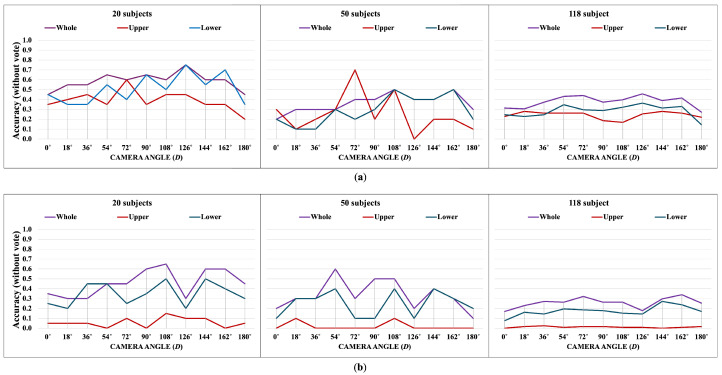
Accuracy of the matching without majority voting on BG condition. (**a**) Accuracy of the joint angles being used as a feature of 20, 50, and 118 subjects. (**b**) Accuracy of the correlation being used as a feature of 20, 50, and 118 subjects.

**Figure 10:**
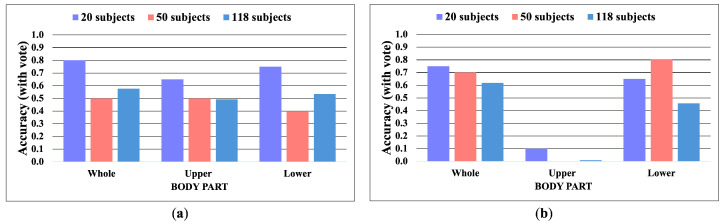
Accuracy of the matching with a majority vote on BG condition. (**a**) Accuracy with majority voting of the joint angles being used as a feature of 20, 50, and 118 subjects. (**b**) Accuracy with majority voting of the correlation being used as a feature of 20, 50, and 118 subjects.

**Figure 11:**
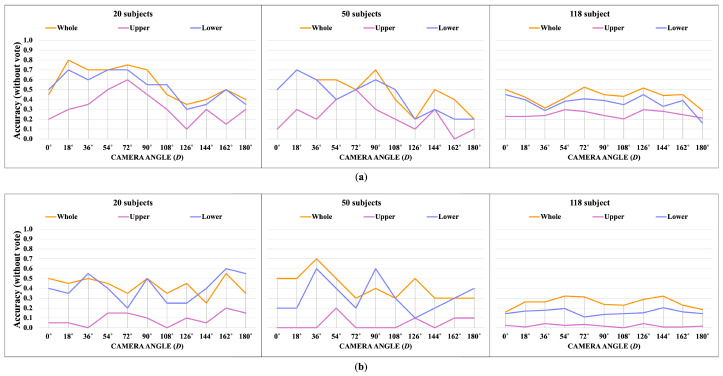
Accuracy of the matching without majority voting on CL condition. (**a**) Accuracy of the joint angles being used as a feature of 20, 50, and 118 subjects. (**b**) Accuracy of the correlation being used as a feature of 20, 50, and 118 subjects.

**Figure 12:**
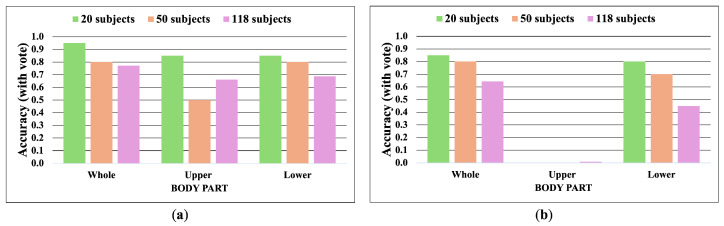
Accuracy of the matching with a majority vote on CL condition. (**a**) Accuracy with majority voting of the joint angles being used as a feature of 20, 50, and 118 subjects. (**b**) Accuracy with majority voting of the correlation being used as a feature of 20, 50, and 118 subjects.

The following Figure 7, Figure 8, Figure 9, Figure 10, Figure 11 and Figure 12 display the accuracy without and with a majority vote on NM, BG, and CL conditions, respectively. The number of subjects for experimentation is 20, 50, and 118 for each condition of CASIA-B. It is important to note that we match the reference and target sequences under identical walking conditions, e.g., matching between two NM sub-datasets. The reason is that the proposed method is pattern matching, and it has no feature learning state. The matching across different walking conditions may decrease the accuracy. Therefore, our focus is to investigate the effect of the walking pattern when the parameters are varied and to improve the multi-view matching based on DTW distance.

The results indicate that a majority vote can enhance the matching performance. It can improve the accuracy by about 30%. This implies that a majority vote can reduce the view variation issue that affects matching. The most reliable feature belongs to the whole body, including both joint angles and correlation features. The findings show that when a person is walking, the whole body is moving together, and it is correlated. Furthermore, the lower body part can be used to identify people by their walking patterns. Unfortunately, the upper body feature achieves the lowest accuracy, especially for the correlation. It is essential to note that the upper body feature consists of two angles, the left and right elbow angles, and this suggests that these two angles are not significant for identification. Naturally, it is difficult to recognize people from only two angles.

The BG sub-dataset captures walkers carrying shoulder bags, and the CL dataset is a scene where subjects walk while wearing a thick outer coat that limits arm movement, which affects the movement of their arms directly. We find that the accuracy of the upper joint angle feature is higher than in the NM condition. This indicates that carrying and clothing conditions make the walking pattern distinct from an identical walking condition. Still, the correlation between upper joint angles remains unreliable.

The overall results imply that the impact of the whole body features is the most significant and reliable, followed by the lower body features.

### 3.2. OUMVLP-Pose Dataset

**Figure 13:**
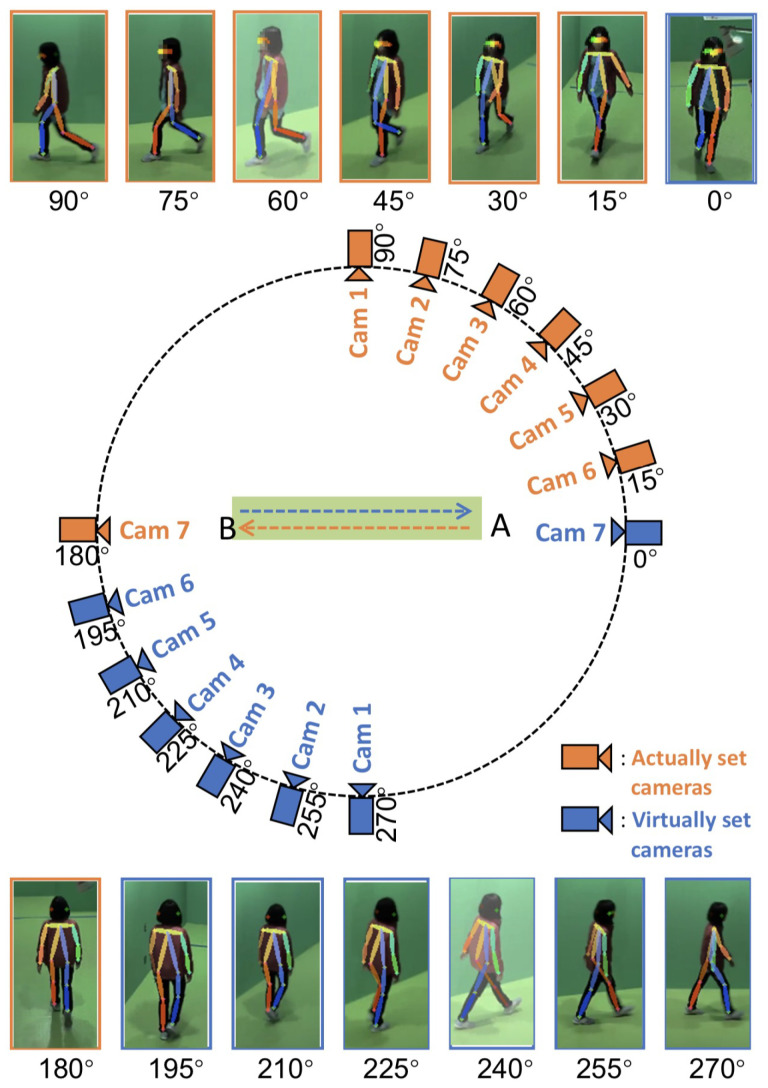
Capturing setup environment of OUMVLP-Pose [33] and sample images with extracted human pose estimation.

OUMVLP-Pose is an OU-ISIR gait database with extracted 2D pose estimation ($p_{i}^{j}\left(x,y,t\right)$) by OpenPose and Alphapose [33]. It contains sequences of 10,307 subjects walking a round trip captured by 14 cameras spanning from $0^{\circ }$ to $270^{\circ }$ with $15^{\circ }$ intervals as shown in Figure 13.

The OUMVLP-Pose dataset provides 18 joint landmarks that are extracted from OpenPose and Alphapose. Unfortunately, it has no foot landmarks provided, and the ankle angles cannot extracted. Thus, the whole-body and lower-body features for this dataset are $\theta^{i,t,D}={\left[\theta^{1,i,t,D}\ldots \theta^{j,i,t,D}\ldots \theta^{6,i,t,D}\right]}^{T}$ and $\theta_{l}^{i,t,D}={\left[\theta^{3,i,t,D}\theta^{4,i,t,D}\ldots \theta^{j,i,t,D}\ldots \theta^{10,i,t,D}\right]}^{T}$, respectively. We apply the proposed method with 20, 50, and 100 subjects from the OUMVLP-Pose dataset.

**Figure 14:**
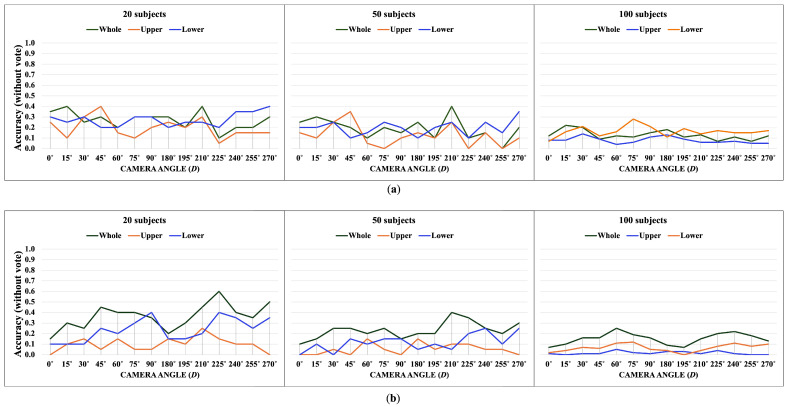
Accuracy of the matching without a majority vote on OUMVLP-Pose (OpenPose). (**a**) Accuracy of the joint angles being used as a feature of 20, 50, and 100 subjects. (**b**) Accuracy of the correlation being used as a feature of 20, 50, and 100 subjects.

**Figure 15:**
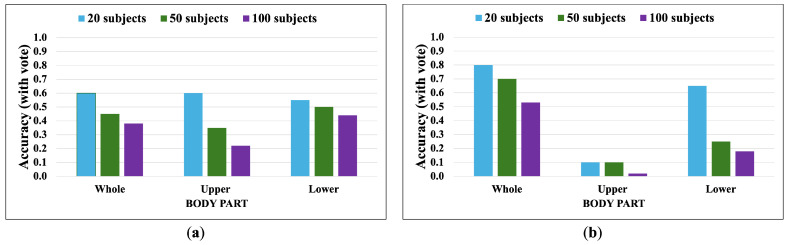
Accuracy of the matching with a majority vote on OUMVLP-Pose (OpenPose). (**a**) Accuracy with majority voting of the joint angles being used as a feature of 20, 50, and 100 subjects. (**b**) Accuracy with majority voting of the correlation being used as a feature of 20, 50, and 100 subjects.

**Figure 16:**
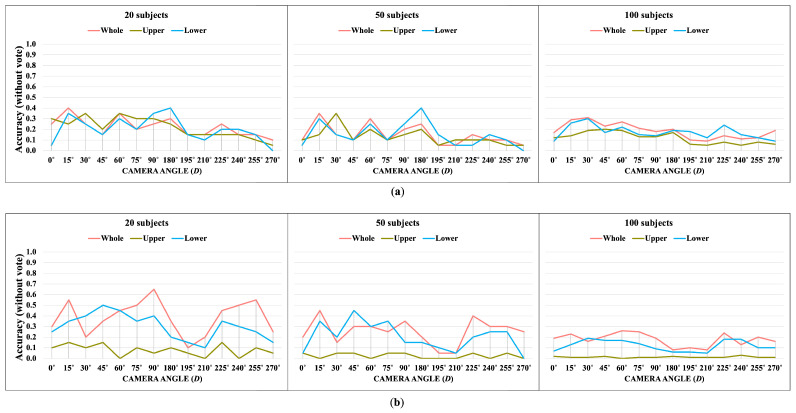
Accuracy of the matching without a majority vote on OUMVLP-Pose (Alphapose). (**a**) Accuracy of the joint angles being used as a feature of 20, 50, and 100 subjects. (**b**) Accuracy of the correlation being used as a feature of 20, 50, and 100 subjects.

**Figure 17:**
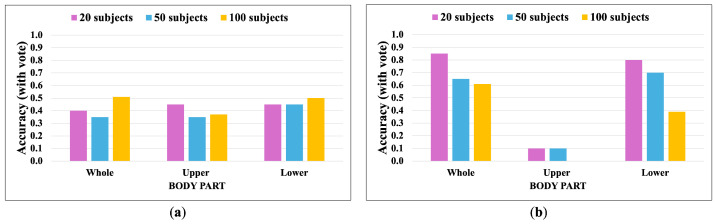
Accuracy of the matching with a majority vote on OUMVLP-Pose (Alphapose). (**a**) Accuracy with majority voting of the joint angles being used as a feature of 20, 50, and 100 subjects. (**b**) Accuracy with majority voting of the correlation being used as a feature of 20, 50, and 100 subjects.

Figure 14, Figure 15, Figure 16 and Figure 17 show the accuracy without and with a majority vote of the OUMVLP-Pose dataset, which provides 2D human pose coordinates extracted by OpenPose and Alphapose. The results suggest that the proposed method can enhance person recognition even when the joint coordinates are 2D and there are no ankle angles. We can achieve an accuracy of about 0.8 after applying a majority vote. Additionally, the data of some subjects are incomplete, and it is a crucial part of this study because it needs two sub-datasets to be a reference and target for the matching. If one is missing, it has no data to match.

The experimental results suggest the correlation between joint angles brings stability to the matching according to the trend of results from all datasets is similar. The upper joint angles feature can identify the identities, unlike correlation. Additionally, an increase in the number of subjects reduces the overall accuracy in matching based on DTW distance. This suggests that our proposed method is suitable for smaller datasets.

The lower body part is sufficient for identifying identities based on a walking pattern. However, the whole body part is the most essential for the correlation feature, and it can be a crucial part of expanding the gait analysis to other tasks.

### 3.3. Execution Time

Table 2 shows the execution time for calculating the joint angles and matching based on DTW distance on the Google Colab’s CPU. The measured time is per ten frames of a single person, and it excludes the time for human pose estimation. The overall time for calculating all tasks for one person per frame is 28.72 ms. The proposed method requires no training phase and can be used with a small amount of data, which can be executed on a CPU. It is a simple and effective method to identify individuals.

### 3.4. Comparative Results with Previous Studies

This section presents the comparative results on the NM condition of the CASIA-B dataset. Table A1 and Table A2 in Appendix A present the original results from [19,34], respectively. We do not intend to compare our results with the original results from their papers, but it is for reference.Both studies show the results of experimenting with separated features. Table 3 presents the results of our experiments, and Table 4 presents the results of the employed CLTS with 20 subjects. The CLTS is a training-based approach, and in it, every sub-dataset from CASIA-B was employed as train and test sets, while we employed only two sub-datasets of the NM condition, which are NM01 as a reference and NM02 as a target for matching. Since the two approaches are formed differently, it is difficult to experiment under identical conditions. For the most accurate implementation, we trained the data for CLTS with the same method as clarified in their paper, but we selected subject numbers 1–21 instead (excluded subject number 5 as specified in their work). For the test set, we employed subject numbers 21–41 of NM01.

Table 3 shows the accuracy with a majority vote on the NM walking condition identical view case of the CASIA-B dataset. Since our method focuses on the integration of different views by voting to overcome the view variation, we neglect the cross-view situation in this experiment. As mentioned previously, our method with a majority vote is suitable for a smaller dataset. Meanwhile, the CLTS achieves a result of about 60% when the dataset is reduced. This implies that the training-based approach requires a large amount of data to increase the accuracy of identification.

In brief, the training-based method requires more data for DNN to learn the features, and it requires a GPU to perform the tasks. The CLTS is an appearance-based approach that uses high-dimensional features for DNN to learn. The experiments were performed on the NVIDIA GeForce GTX 1080Ti GPU. Additionally, PoseGait used a Tesla K80 GPU to perform 2D pose estimation and feature extraction based on CNN. While the proposed method performed the tasks on the CPU of Google Colab, additionally, the proposed method does not require a re-training process when adding or deleting identities. It makes the proposed method faster and lighter to implement and preserves high efficiency.

## 4. Conclusions

This study presents multi-view gait recognition by majority voting based on the features of human body parts. We analyze gait by calculating joint angles and their correlation. We divide features into three parts, i.e., whole, upper, and lower body, to study the impact of different body parts on gait analysis. DTW employs these features to match people in separate multiple cameras. Then, we apply a majority vote to integrate the separated data to improve the accuracy and test the experiment over three different walking conditions in the CASIA-B and OUMVLP-Pose datasets. Furthermore, we divide the number of subjects into 20, 50, and 118 subjects for the CASIA-B dataset, and 20, 50, and 100 subjects for the OUMVLP-Pose dataset to observe the trend of the matching accuracy when the amount of data is varied.

According to the findings, integrating the view variations by majority voting can enhance the accuracy of the matching based on DTW distance to 30% compared to the case without a majority vote. We find that the features related to the lower body are sufficient for identifying people using the joint angle. However, the whole body is crucial for other tasks of gait analysis, such as detecting emotions and predicting one’s health. In this case, the correlation feature adds more reliability to the results. The proposed method is suitable for identifying identities with a smaller database. Additionally, the availability of data visualization enables one-by-one detailed analyses, which is advantageous for the expansion of our future tasks. Furthermore, it can be executed on the CPU according to a no-training state. Thus, the GPU and complicated environment are unnecessary, leading to reductions in both cost and time.

## Figures and Tables

**Table 1:** Sample of the calculated individual correlation between each joint angle.

	LElbow	RElbow	LHip	RHip	LKnee	RKnee	LAnkle (Front)	RAnkle (Front)	LAnkle (Back)	RAnkle (Back)
**LElbow**	**1.00**	**0.43**	**−0.47**	**−0.32**	**−0.65**	**−0.30**	**−0.31**	**−0.01**	**0.10**	**0.03**
**RElbow**	**0.43**	**1.00**	**−0.37**	**0.06**	**−0.19**	**0.17**	**−0.34**	**−0.46**	**0.24**	**0.54**
**LHip**	**−0.47**	**−0.37**	**1.00**	**0.06**	**0.58**	**0.28**	**0.07**	**−0.08**	**−0.23**	**−0.30**
**RHip**	**−0.32**	**0.06**	**0.06**	**1.00**	**0.33**	**0.86**	**−0.33**	**−0.54**	**0.04**	**0.45**
**LKnee**	**−0.65**	**−0.19**	**0.58**	**0.33**	**1.00**	**0.47**	**0.30**	**−0.34**	**−0.52**	**0.08**
**RKnee**	**−0.30**	**0.17**	**0.28**	**0.86**	**0.47**	**1.00**	**−0.43**	**−0.61**	**−0.13**	**0.41**
**LAnkle** **(Front)**	**−0.31**	**−0.34**	**0.07**	**−0.33**	**0.30**	**−0.43**	**1.00**	**0.23**	**−0.31**	**0.00**
**RAnkle** **(Front)**	**−0.01**	**−0.46**	**−0.08**	**−0.54**	**−0.34**	**−0.61**	**0.23**	**1.00**	**0.13**	**−0.57**
**LAnkle** **(Back)**	**0.10**	**0.24**	**−0.23**	**0.04**	**−0.52**	**−0.13**	**−0.31**	**0.13**	**1.00**	**0.17**
**RAnkle** **(Back)**	**0.03**	**0.54**	**−0.30**	**0.45**	**0.08**	**0.41**	**0.00**	**−0.57**	**0.17**	**1.00**

**Table 2:** Execution time for calculating joint angles and DTW matching per 10 frames of one person on the virtual CPU of Google Colab.

Joint Angles Calculation	DTW (Joint Angles)	DTW (Correaltion)
3.22 ms	182 ms	102 ms

**Table 3:** Accuracy with a majority vote (%) on NM walking condition of the CASIA-B dataset (ours).

		Accuracy with a Majority Vote (%)		
		20 Subjects	50 Subjects	118 Subjects
Joint angles	Whole	80.00	80.00	68.64
	Upper	55.00	52.00	50.00
	Lower	75.00	74.00	55.93
Correlation	Whole	85.00	78.00	65.25
	Upper	10.00	8.00	2.45
	Lower	80.00	62.00	39.83

**Table 4:** Average rank-1 accuracy (%) on NM walking condition of the CASIA-B dataset (CLTS).

	20 Subjects
Include identical view	62.83
Exclude identical view	59.14

## Data Availability

Restrictions apply to the availability of these data. Data were obtained from The Institute of Automation, Chinese Academy of Sciences (CASIA) and The Institute of Scientific and Industrial Research (ISIR), Osaka University (OU), and are available at http://www.cbsr.ia.ac.cn/english/Gait%20Databases.asp (accessed on 3 January 2024) and http://www.am.sanken.osaka-u.ac.jp/BiometricDB/GaitLPPose.html (accessed on 3 January 2024) with the permission of CASIA and ISIR, respectively.

## Appendix A

**Table A1:** Averaged rank-1 accuracy (%) of multi-scale temporal features on NM condition of the CASIA-B dataset from CLTS. The check mark indicates the features used to evaluate this method’s efficiency [34].

Multi-Scale Features			Rank-1 Accuracy
Frame-Level	Short-Term	Long-Term	NM
√			96.9
	√		97.2
		√	95.9
√	√		97.0
√		√	97.4
	√	√	97.4
√	√	√	**97.8**

Bold represents the maximum Rank-1 accuracy.

**Table A2:** Recognition rates (%) of different features on NM condition of the CASIA-B dataset from PoseGait [19].

Features	Recognition Rates
$\left[f_{pose}\right]$	60.92
$\left[f_{angle}\right]$	46.97
$\left[f_{limb}\right]$	42.40
$\left[f_{motion}\right]$	48.95
$\left[f_{pose},f_{angle},f_{limb},f_{motion}\right]$	**63.78**

Bold represents the maximum recognition rates.
